# Beyond Passive Immunity: Three Neonatal Influenza Cases Highlighting Impact of Missed Maternal Vaccination

**DOI:** 10.3390/clinpract15070124

**Published:** 2025-06-30

**Authors:** Irina Profir, Cristina-Mihaela Popescu, Gabriel Valeriu Popa, Aurel Nechita

**Affiliations:** 1 Clinical Medical Department, Faculty of Medicine and Pharmacy, “Dunărea de Jos” University of Galați, 800216 Galati, Romania; irina.profir@ugal.ro (I.P.); nechitaaurel@yahoo.com (A.N.); 2 “Sf. Ioan” Clinical Emergency Pediatric Hospital in Galați, 800487 Galati, Romania; 3 Dental-Medicine Department, Faculty of Medicine and Pharmacy, “Dunărea de Jos” University of Galați, 800201 Galati, Romania; gabriel.popa@ugal.ro

**Keywords:** infant, respiratory tract infections, immunization

## Abstract

**Background**: Neonatal influenza is a rare condition. Young infants have immature immune defenses and are unable to receive direct vaccination; this can result in significant illness. Maternal anti-influenza immunization during pregnancy provides passive antibodies to the newborn via transplacental transfer, significantly decreasing the incidence and severity of influenza in early infancy. Nevertheless, the vaccination coverage during pregnancy remains low in many regions, leaving certain neonates without adequate protection. **Methods**: We present three cases of laboratory-confirmed influenza infection in neonates admitted to the “Sf. Ioan” Clinical Emergency Pediatric Hospital in Galați and conduct a literature review. The clinical presentation, co-infections, timing of antiviral therapy, laboratory findings, maternal vaccination status, and outcomes (including the hospitalization duration and recovery) were systematically analyzed for each case. **Results**: All three neonates were full-term and previously healthy, born to mothers who had not received influenza vaccinations during their pregnancies. They presented at ages ranging from 2 to 4 weeks with fever, respiratory symptoms including a cough, nasal congestion, and respiratory distress, as well as feeding difficulties. One case involved a co-infection with Bordetella pertussis, which manifested as a severe paroxysmal cough, cyanosis, and apnea. Laboratory findings in the cases with influenza alone indicated leukopenia accompanied by normal C-reactive protein levels. In the co-infection case, leukocytosis, lymphocytosis, and thrombocytosis were observed. All the infants received oseltamivir treatment within 48 h of the symptom onset; the case with pertussis co-infection also received azithromycin. Each infant required supplemental oxygen, but none necessitated mechanical ventilation. Clinical improvement was observed in all cases, with hospitalization ranging from 6 to 7 days and complete recovery without complications. **Conclusions**: Neonatal influenza may result in considerable morbidity, particularly in infants born to unvaccinated mothers. Positive outcomes, however, have been correlated with early diagnosis and antiviral treatment. Pertussis co-infection may exacerbate clinical progression, underscoring the importance of maternal immunization against both influenza and pertussis. In this case series, we aim to present three cases of laboratory-confirmed influenza in neonates born to mothers who were not immunized against influenza during pregnancy. These cases highlight the clinical presentations of neonatal influenza, underscore the risks associated with pertussis co-infection, and reinforce the importance of maternal influenza and Tdap vaccination for preventing severe outcomes in newborns.

## 1. Introduction

Influenza infections in neonates, although relatively uncommon, present considerable health risks due to the underdeveloped immune systems of newborns [1]. Young infants have immature immune defenses and are not eligible for direct influenza vaccination, as the current vaccines are not approved for use in infants under six months of age [2]. Maternal immunization during pregnancy is crucial because it confers passive immunity to infants, thereby reducing the incidence of laboratory-confirmed influenza and associated hospitalizations during early infancy [3,4,5,6]. A recent case–control study revealed 53% effectiveness in preventing influenza illness in infants under three months when maternal immunization was performed during the third trimester [3]. Despite these benefits, the vaccination rates among pregnant women remain suboptimal because of barriers such as vaccine hesitancy, a lack of awareness, and limited access [7,8,9,10,11].

This case series explores the clinical and public health consequences of low maternal influenza and pertussis (Tdap) vaccination coverage, illustrated through three neonatal cases admitted to the “Sf. Ioan” Clinical Emergency Pediatric Hospital in Galați and supported by a literature review. The study was conducted in accordance with the Declaration of Helsinki and approved by the Ethics Committee of the “Sf. Ioan” Clinical Emergency Pediatric Hospital in Galați (protocol code 23183/24, September 2024; renewed as 7851/10, April 2025). In this case report, we aim to present three cases of laboratory-confirmed influenza in neonates born to mothers who were not immunized against influenza during pregnancy. All the infants were hospitalized at the “Sf. Ioan” Clinical Emergency Pediatric Hospital in Galați. These cases highlight the clinical presentations of neonatal influenza, underscore the risks associated with pertussis co-infection, and reinforce the importance of maternal influenza and Tdap vaccination for preventing severe outcomes in newborns.

## 2. Case Series

Case 1: A 14-day-old male neonate, who was born at thirty-eight weeks of gestation through spontaneous vaginal delivery with a birth weight of 3100 g, presented with a one-day history of high fever, peaking at 39 °C, poor feeding, a dry cough, and nasal congestion. The infant was mixed-fed. Upon examination, their heart rate was 170 beats per minute, and their respiratory rate was 55 breaths per minute. The patient was febrile and exhibited mild respiratory distress, with harsh breath sounds and occasional rhonchi noted during auscultation. Their oxygen saturation levels fluctuated between 95% and 98% in room air, with intermittent desaturations occurring during coughing episodes, necessitating supplemental oxygen at a rate of 4 to 6 L per minute administered via a headbox. A rapid antigen test for influenza yielded a positive result for Influenza B, which was later confirmed by a reverse transcription polymerase chain reaction (RT-PCR). The laboratory findings included a leukocyte count of 4.3 × 10^3^/µL, which indicated lymphopenia at 1.26 × 10^3^/µL, and a C-reactive protein (CRP) level below 0.5 mg/dL. The mother of the infant exhibited influenza-like symptoms concurrently and had not received an influenza vaccination during her pregnancy. The neonate was admitted to the hospital and received oseltamivir treatment (3 mg/kg per dose, administered twice daily for five days), accompanied by supportive care (antipyretics, hydration, and oxygen therapy). At no time during hospitalization did the patient exhibit unresponsiveness or impaired consciousness. The infant demonstrated steady improvement and was discharged after a six-day hospitalization.

Case 2: A 15-day-old female neonate, who was born at term via spontaneous vaginal delivery with a birth weight of 3200 g, was admitted with a one-day history of fever (38.9 °C), difficulties in feeding, a dry cough, and nasal congestion. The physical examination revealed a heart rate of 213 beats per minute, a respiratory rate of 60 breaths per minute, and signs of mild respiratory distress. Auscultation indicated the presence of mild rhonchi; the infant’s oxygen saturation levels were recorded at 94–98% in room air, with occasional declines during episodes of coughing, necessitating brief administrations of supplemental oxygen (4–6 L/min). Rapid testing yielded a positive result for Influenza A, which was confirmed through an RT-PCR. The mother had reported flu-like symptoms at around the same time and was unvaccinated against influenza. Blood tests showed a leukocyte count of 5.2 × 10^3^/µL and a CRP level of less than 0.5 mg/dL. The infant received oseltamivir (3 mg/kg twice daily for 5 days) in addition to supportive care. At no time during hospitalization did the patient exhibit unresponsiveness or impaired consciousness. Her condition improved progressively throughout the treatment period, and she was discharged following a 6-day hospitalization.

Case 3: A 27-day-old male neonate, born at term via vaginal delivery (birth weight of 3400 g), presented with a 2–3 day history of feeding difficulties, a paroxysmal cough, apnea episodes, cyanosis, and fever (peak temperature of 39 °C). On examination, he appeared ill with a heart rate of 150 beats/min and a respiratory rate of 50 breaths/min. His oxygen saturation fluctuated between 89% and 98% in room air. He had signs of moderate respiratory distress and scattered crackles on lung auscultation. During coughing episodes, he exhibited apnea with transient unresponsiveness and oxygen desaturation, requiring supplemental oxygen (4–6 L/min administered via a mask/headbox). The RT-PCR result for a nasopharyngeal sample was positive for Influenza B and *Bordetella pertussis*. His laboratory workup showed an elevated white blood cell count of 27.98 × 10^3^/µL, with an absolute lymphocyte count (ALC) of 17.2 × 10^3^/µL, consistent with pertussis-associated lymphocytosis. His platelet count was also elevated (553 × 10^3^/µL). The infant’s inflammatory markers remained low [C-reactive protein (CRP) level of <0.5 mg/dL, procalcitonin (PCT) level of 0.176 ng/mL]. The infant was admitted to isolation and treated with oseltamivir (3 mg/kg twice daily for 5 days) and azithromycin (10 mg/kg/day for 5 days) in addition to supportive care (oxygen supplementation and fluid support). Gradual clinical improvement was observed over the next few days. He was weaned off oxygen by day 5 and discharged after 7 days of hospitalization. This patient’s mother had not received influenza or Tdap vaccines during pregnancy.

All three neonates underwent comprehensive microbiological testing upon admission. Nasal and pharyngeal swabs were cultured for *Staphylococcus aureus* and β-hemolytic *Streptococcus*. In addition, multiplex PCR testing was performed to detect a wide range of respiratory pathogens, including SARS-CoV-2, Influenza A and B, respiratory syncytial virus (RSV), human adenovirus, human metapneumovirus, enterovirus, parainfluenza virus types 1–4, human bocavirus (types 1–4), human rhinovirus (types A/B/C), and seasonal human coronaviruses (229E, NL63, OC43). The presence of atypical and bacterial pathogens such as *Chlamydophila pneumoniae*, *Mycoplasma pneumoniae*, *Legionella pneumophila*, *Bordetella pertussis*, *Bordetella parapertussis*, *Streptococcus pneumoniae*, and *Haemophilus influenzae* was also evaluated. Aside from the influenza virus and *Bordetella pertussis* in Case 3, no other pathogens were detected. Each patient was promptly isolated upon admission, and standard sterilization and infection control protocols were strictly followed throughout their hospitalization.

The bloodwork at admission and discharge for all three patients is provided in Table 1.

## 3. Discussion

Neonatal influenza represents a rare diagnosis. However, our cases demonstrate that young infants are susceptible to becoming considerably ill, particularly when maternal immunity is deficient.

Neonates have clinical manifestations that may vary and are frequently nonspecific [12]. In our three cases, fever was the main symptom, accompanied by respiratory symptoms, including a cough, nasal congestion, and tachypnea. A recent study involving 21 neonates diagnosed with influenza reported that 71% exhibited symptoms of fever, while more than 60% experienced difficulties in feeding [1]. Approximately 29% of the infants in that study only presented a cough or nasal congestion [1]. This variability emphasizes that influenza in infants can resemble other neonatal infections. Severe apnea and cyanosis, as observed in Case 3, may occur and could initially imply diagnoses such as sepsis or pertussis. Indeed, influenza in this age group may manifest with apnea or even a sepsis-like presentation in the absence of elevated fever [13].

Clinicians should maintain an increased suspicion of viral respiratory infections in neonates presenting with these symptoms, particularly during the influenza season or in the presence of ill contacts within the home [1].

Co-infections among young infants are relatively common and have the potential to exacerbate the clinical progression of neonatal influenza. Research indicates that nearly 50% of infants hospitalized with pertussis experience a concurrent viral infection, which includes occasional cases of co-infection with the influenza virus [14].

In our case, the dual infection with *Bordetella pertussis* and influenza was associated with a slightly longer hospital stay (7 days compared to 6 in the other two cases). However, we cannot establish a causal relationship between co-infection and the disease severity or hospitalization duration based on a single case. The impact of viral–bacterial co-infections on clinical outcomes remains a topic of ongoing debate. In a 2011 study published by Martin-Loeches et al., Cox regression adjusted for confounders did not confirm a statistically significant association between the mortality in intensive care units and co-infection [15]. A systematic review by Qiao et al. showed that unadjusted analyses indicated higher mortality with co-infections [16]. Thus, although co-infections may influence the disease course, further robust, controlled studies are needed to clarify their true impact.

At our institution, the availability of rapid influenza diagnostic tests and PCR confirmation facilitated prompt diagnosis and treatment. This is the standard practice during the influenza season, which involves initial screening with rapid antigen diagnostic tests, followed by confirmatory RT-PCR testing. This two-step approach optimizes the timely identification of influenza and enables us to investigate potential co-infections using multiplex PCR assays, particularly in cases of severe or atypical presentations. For practitioners without routine access to PCR diagnostics, we recommend using rapid antigen tests as an initial assessment during influenza season, followed by referral to or collaboration with facilities that offer PCR confirmation and broader multiplex panels when the clinical severity or complexity suggests co-infection or uncertainty in the diagnosis.

The prognosis is generally favorable if the infection is recognized early and managed appropriately [1]. In this case series, the disease severity was assessed based on clinical features, including respiratory distress, oxygen requirements, and recovery trajectories, with all the cases reflecting a moderate illness severity without progression to respiratory failure.

The laboratory findings in our cases offer further insight into the particularities of neonatal influenza infections. Cases 1 and 2 exhibited leukopenia (4.3 and 5.2 × 10^3^/µL, respectively) and lymphopenia (ALN of 1.26 × 10^3^/µL and 1.68 × 10^3^/µL, respectively) during the acute phase. Neonates affected by influenza may present with normal or only mildly altered white blood cell counts. In a Chinese case series investigating neonatal influenza, the majority of infants (86%) displayed normal white blood cell counts, and none exhibited significant neutrophilia, which aligns with a primarily viral etiology [1]. However, pediatric data indicate that leukopenia, particularly lymphopenia, is a prevalent response to influenza infection [17]. A low lymphocyte count has been increasingly recognized as a marker of severe viral infections, including influenza, even in neonatal populations. Studies have demonstrated that the lymphocyte count serves as a predictor of the disease severity in children with influenza, with lower lymphocyte counts correlating with worse outcomes [18,19]. One study noted an ALC of <1500/μL at presentation as a significant predictor of progression to ARDS in influenza patients [20]. Our findings of leukopenia and lymphopenia are consistent with recent pediatric studies, which report these as common hematologic patterns in children with influenza [21].

The cases presenting solely with influenza demonstrated reduced white blood cell counts and lymphopenia. However, our third patient exhibited marked leukocytosis (~28 × 10^3^/µL) with lymphocytosis (17.2 × 10^3^/µL), which aligns with the typical hematologic findings in pertussis [22,23]. While pertussis in infants is typically associated with hyperleukocytosis, the link between elevated white cell counts and a prolonged hospital stay is not definitively causal. Numerous studies have observed that extreme leukocytosis, especially when accompanied by lymphocytic predominance, is associated with severe disease or mortality in infants with pertussis [24,25]. Nonetheless, these studies highlight an association rather than causation, and the outcomes are likely to be influenced by additional factors such as pulmonary hypertension and respiratory compromises [25,26].

Platelet abnormalities are commonly observed in pediatric respiratory infections, including influenza and pertussis. Thrombocytopenia has been reported in a significant proportion of children hospitalized with influenza, and in some cases, it may be followed by reactive thrombocytosis during the acute or recovery phase [27,28]. Severe influenza can occasionally be associated with consumptive coagulopathy or thrombocytopenia, as illustrated in a published case of fulminant neonatal Influenza B, where the platelet count declined to 37 × 10^3^/μL [13]. Although none of our patients exhibited such severe complications, monitoring the platelet trends remains important, as a sudden decline may indicate disseminated intravascular coagulation or secondary sepsis [13]. In contrast, our case of influenza and pertussis co-infection demonstrated thrombocytosis (platelet count of 553 × 10^3^/μL), a known hematologic pattern in pertussis. Thrombocytosis has also been linked to an increased risk of pneumonia and other lower respiratory tract infections in children [28,29].

In this age group, inflammatory markers alone provide limited diagnostic utility due to infants’ immature immune responses [30]. Instead, pathogen-specific diagnostics are essential: rapid multiplex PCR testing enabled the early detection of influenza and, in one case, Bordetella pertussis, facilitating the timely administration of antiviral and antibiotic treatment. Recent studies have demonstrated that performing a point-of-care multiplex PCR in pediatric and neonatal settings significantly improves antimicrobial stewardship by reducing the empiric antibiotic use and enhancing the treatment appropriateness (e.g., of oseltamivir for influenza) without compromising safety [31,32]. All three neonates exhibited low inflammatory markers (CRP of <0.5 mg/dL and a normal PCT), likely reflecting viral infection [30]. Even in Case 3, despite a dual infection, the infant’s CRP levels remained low; pertussis infections do not frequently elicit heightened acute-phase reactants, complicating diagnosis based on inflammatory markers [28].

While the CRP level is often normal or only mildly elevated in typical pertussis cases, recent studies have shown that elevated CRP levels may be associated with more severe presentations, including those requiring ICU admission and exchange transfusion [33,34]. In our series, the CRP level remained low even in the patient with pertussis co-infection, suggesting a milder disease course.

Pediatric studies have employed a PCT threshold of approximately 0.25 ng/mL to guide antibiotic usage, revealing that low levels significantly reduce the likelihood of bacterial co-infection and can safely diminish unnecessary antibiotic exposure [28,35].

The timing of antiviral treatment is of paramount importance in determining neonatal influenza outcomes. In all three cases presented, treatment with oseltamivir was initiated within 1 to 3 days of the symptom onset, immediately upon diagnosis. The infant with pertussis co-infection received targeted therapy for both pathogens (antivirals and appropriate antibiotics). Our cases are consistent with the existing literature, which reports that suitable antiviral therapy and supportive care can lead to survival in the absence of severe comorbidities [1]. The early initiation of antiviral therapy likely played a significant role in the favorable clinical progress and outcome of all our cases [36].

All the patients in our series were administered the standard dosing regimen (3 mg/kg twice daily for five days) and tolerated the therapy well, with no adverse effects reported. This is consistent with recent findings that regard it as safe for neonates and as having the potential to be life-saving in preventing disease progression, particularly in severe cases or during outbreaks [1,37].

Although a prospective pediatric study demonstrated that early oseltamivir administration significantly shortened the clinical duration of influenza illness in infants from approximately 10.6 days to about 3.4 days [38], our neonates required longer hospitalization durations (average of 6–7 days). This discrepancy likely arose from neonatal-specific factors, including immature immune responses, cautious monitoring practices for young infants, the necessity for supportive care (e.g., oxygen therapy and hydration), and the presence of bacterial co-infection in one case. Thus, oseltamivir likely contributed positively to the clinical outcomes; however, neonates typically require extended monitoring and support, which lengthens their hospitalization, irrespective of the antiviral efficacy. The current clinical guidelines advocate for antiviral treatment for both suspected and confirmed cases of influenza in neonates and young infants, as these populations have an elevated risk for complications [39,40].

Our limited experience confirms that timely antiviral therapy, administered within 48 h of the symptom onset, can facilitate rapid improvement and may prevent the need for intensive respiratory support in cases of neonatal influenza.

We highlight that all three infants had been full-term and previously healthy; prematurity and underlying medical conditions can further elevate the risk of severe influenza outcomes in neonates [1].

Despite the serious nature of their illnesses, all three neonates in this series exhibited favorable outcomes, with hospital stays averaging one week and complete recoveries. None of the infants developed comorbid pneumonia or experienced respiratory failure necessitating mechanical ventilation, and no long-term complications were observed at the time of discharge. Early diagnosis and timely supportive care are likely contributors to the prevention of disease progression. Published reports indicate that the outcomes associated with neonatal influenza vary widely, ranging from mild outpatient cases to severe pneumonia with multi-organ failure [13].

A crucial common denominator in our cases was the maternal vaccination status. All three infants were born to mothers who had not received influenza vaccination during pregnancy. Additionally, the mother of the infant co-infected with pertussis had not received a Tdap vaccine.

Neonatal immune protection arises from hemagglutinin-specific IgG that concentrates in the cord blood [41,42], ensuring elevated antibody titers in infants [43]. A lack of maternal antibodies may explain why our patients, all under one month old, contracted influenza and required hospitalization, as maternal antibodies might have prevented or attenuated these infections in our cases [1,44].

Maternal immunization against influenza is well documented as mitigating influenza illnesses in young infants [3,45,46]. Randomized trials and observational studies consistently indicate a fifty percent or more reduction in infant influenza when vaccinating pregnant women [6,47]. Furthermore, another multi-season study estimated that maternal influenza vaccination decreased the risk of influenza-associated hospitalization in infants under six months by approximately one-third overall and by more than fifty percent in infants under three months [3].

Nevertheless, the uptake remains suboptimal, as only approximately 47% of pregnant women reported receiving flu vaccination in a recent US survey, partly due to enduring hesitancy and access barriers [9,10,11]. These challenges are not limited to the U.S.; a systematic review of European studies by Adeyanju et al. (2021) identified multiple determinants of maternal influenza vaccine hesitancy, including safety concerns, a lack of healthcare provider recommendations, limited knowledge, and mistrust in health authorities [48]. Similarly, recent reports from Spain and France revealed even lower influenza vaccine coverage among pregnant women—only 6.8–11.9% in Catalonia, Spain, between 2015 and 2018, and 59% of pregnant women in France reported never being offered the vaccine—further underscoring the variability and vulnerability of the maternal immunization uptake across high-income settings [48,49,50]. Addressing these gaps by reinforcing the safety and efficacy of maternal immunization and emphasizing its neonatal benefits is critical to enhancing infant influenza outcomes [4,51,52,53].

Similarly, maternal Tdap vaccination during pregnancy remains the primary strategy to protect young infants from pertussis; unvaccinated mothers leave their infants vulnerable to severe pertussis until the infant’s own immunizations commence. In alignment with this evidence, the World Health Organization, along with clinical guidance from maternal health experts, strongly advocates for influenza vaccination at any point during pregnancy [53,54].

Steinhoff et al. demonstrated that maternal vaccination during periods of active influenza circulation reduced infant respiratory illnesses with fever by nearly 50% and improved the birth outcomes (fewer small-for-gestational-age infants and higher birth weights) [55]. This provides indirect evidence of mitigated postnatal disease severity. Complementing this, a meta-analysis integrating observational and randomized controlled trial data established 63% efficacy (95% CI: 5–85%) against laboratory-confirmed influenza in infants under 6 months of age [56]. Furthermore, a 2018 meta-analysis study of neonates under 6 months reported a 34% reduction (95% CI: 12–50%) in influenza-associated hospitalizations or emergency department visits overall, with the efficacy rising to 53% among infants under 3 months [45]. This highlights how maternal antibodies mitigate disease severity and reduce the risk of hospitalization.

In contrast, our three neonates, all lacking maternal vaccination, developed significant febrile respiratory distress necessitating hospitalization, with one case complicated by severe pertussis co-infection. These infants derived no benefit from passive immunity conferred by antenatal vaccination. Our findings, although limited, align with the existing literature, indicating that maternal immunization not only decreases the disease incidence but also ameliorates the clinical severity in infants who contract influenza. This comparative analysis highlights the protective advantages of maternal vaccination and reinforces our advocacy for robust immunization implementation during pregnancy.

This case series underscores the significance of considering co-infections in any critically ill neonate with respiratory disease. Additionally, it reinforces the existing recommendations for maternal Tdap vaccination during every pregnancy, along with the need for family members to be up to date with their pertussis and influenza vaccinations [57,58].

Moreover, surveys conducted in Europe and Australia indicate that many pregnant women continue to forgo influenza or Tdap vaccines due to misconceptions regarding their safety and low levels of awareness [7,48,57,59].

Despite its strong recommendations and proven benefits, the rates of maternal influenza and pertussis vaccination remain low globally, often below 50%, due to a combination of misconceptions and access issues. Common safety concerns persist: many expectant mothers fear that vaccination could harm the fetus or trigger pregnancy complications, despite the lack of evidence to support these fears [60,61,62,63,64,65,66,67,68].

Knowledge gaps also exist, even when vaccines are available; a study from Greece revealed that only ~25% of pregnant women felt adequately informed about the flu and Tdap vaccines [65]. Another review reported that a lack of provider recommendations, logistical barriers (e.g., missed antenatal opportunities, limited clinic availability), and demographic factors (including a younger age, ethnic minority status, or lower socioeconomic status) are strongly associated with the underutilization of immunization services [64]. Digital misinformation also remains influential, shaping negative perceptions of vaccine safety and efficacy [62,66].

Our case series underscores the dire consequences of these barriers: all the mothers were unvaccinated despite clear antenatal care guidelines, and their infants required hospitalization for influenza, with one severe co-infection by pertussis. To improve the maternal vaccination rates, we advocate for multifaceted strategies, including consistent and strong recommendations from prenatal care providers, integrating immunizations into routine antenatal visits, providing tailored education that addresses common myths, and combating online misinformation with trusted, evidence-based resources.

While limited to three cases, this series includes all the neonatal influenza cases hospitalized at the “Sf. Ioan” Clinical Emergency Pediatric Hospital in Galați between 2018 and 2025, all clustered in February 2025. Another limitation of our series is the lack of maternal antibody titers, which would highlight minimal maternal protection due to a lack of maternal immunization. However small our sample was, these cases underscore the importance of vigilance and prompt intervention in achieving favorable outcomes for neonatal influenza.

We highlight the consequences of missed maternal vaccinations, developing influenza and acquiring pertussis, which can result in a more complicated illness. Increasing the maternal vaccination rates through education and public health initiatives could have averted these neonatal illnesses and the subsequent hospitalizations [3,54].

Healthcare providers caring for pregnant women and newborns should advocate vigorously for maternal immunizations as a standard practice in prenatal care, which will consequently protect newborns in those vulnerable initial months of life.

## 4. Conclusions

Considerable morbidity can occur from influenza infections in neonates, especially in the absence of maternal immunity. Our case series illustrates that even healthy, full-term infants may experience significant febrile illness and respiratory distress requiring hospitalization. Co-infection with Bordetella pertussis can further complicate clinical presentations, emphasizing the need for comprehensive multiplex testing in critically ill neonates.

Timely diagnosis using rapid influenza diagnostics, followed by the prompt initiation of antiviral therapy, such as oseltamivir, is critical in this vulnerable population and directly contributed to positive outcomes in our cases. Clinicians must maintain an awareness that oseltamivir is safe and effective in neonates and should not hesitate to initiate treatment when influenza is suspected or confirmed. Most critically, our cases underscore the indispensable role of maternal immunization in preventing neonatal influenza and its associated complications. Public health campaigns targeting vaccine hesitancy and emphasizing neonatal protection are urgently needed.

## Figures and Tables

**Table 1:** Bloodwork at admission and discharge for all three patients.

Parameter	Reference Values	Patient 1		Patient 2		Patient 3	
		Admission	Discharge	Admission	Discharge	Admission	Discharge
Hb (g/dL)	15–24	13.2	11.8	12.6	11	11.4	10.5
HCT (%)	41–70	39.8	34.6	37.4	34.2	35.4	31.9
RBC (10^6^/µL)	4.1–6.7	3.86	3.6	3.6	3.58	3.68	3.37
WBC (10^3^/µL)	6–17.5	4.3	10.9	5.2	11	27.98	11.36
ANC (10^3^/µL)	1.8–5.4	2.26	2.1	2.2	5.4	5.13	1.42
ALC (10^3^/µL)	2.8–9	1.26	6.8	1.69	4.2	17.19	7.75
AMC (10^3^/µL)	0–1.7	0.61	1.87	1.29	0.8	4.57	1.82
PLT (10^3^/µL)	220–520	204	477	261	452	553	446
CRP (mg/dL)	0–0.5	<0.50	<0.50	<0.50	<0.50	<0.50	<0.50
PCT (ng/mL)	0–0.5	0.237	-	0.245	-	0.176	-
Creatinine (mg/dL)	0.2–0.4	0.41	0.35	0.33	0.4	0.3	0.27
AST (U/L)	17–59	53	46	57–42	42	41	46
ALT (U/L)	4–44	35	30	29–30	30	34	38
CK (U/L)	60–305	138	-	142	-	110	-
LDH (U/L)	125–735	296	-	422	-	378	-
Urea (mg/dL)	11–45	6.9	7.5	20.4	10.7	28	25

[Hb—hemoglobin; HCT—hematocrit; RBCs—red blood cells; WBCs—white blood cells; ANC—absolute neutrophil count; ALC—absolute lymphocyte count; AMC—Absolute monocyte count; PLT—platelet count; CRP—C-reactive protein; PCT—procalcitonin; AST—aspartate aminotransferase; ALT—alanine transaminase; CK—creatine kinase; LDH—lactate dehydrogenase].

## Data Availability

The original contributions presented in this study are included in the article. Further inquiries can be directed to the corresponding author.

## Abbreviations

The following abbreviations are used in this manuscript:

RT-PCR	Reverse transcription polymerase chain reaction
ARDS	Acute respiratory distress syndrome
Hb	Hemoglobin
HCT	Hematocrit
RBC	Red blood cell
WBC	White blood cell
ANC	Absolute neutrophil count
ALC	Absolute lymphocyte count
AMC	Absolute monocyte count
PLT	Platelet count
PCT	Procalcitonin
AST	Aspartate aminotransferase
ALT	Alanine transaminase
CRP	C-reactive protein
CK	Creatine kinase
LDH	Lactate dehydrogenase
Tdap	Tetanus, diphtheria, and pertussis
